# EZH2 inhibition in *ARID1A* mutated clear cell and endometrioid ovarian and endometrioid endometrial cancers

**DOI:** 10.1186/s40661-017-0052-y

**Published:** 2017-10-31

**Authors:** Jill K. Alldredge, Ramez N. Eskander

**Affiliations:** 1 University of California, 101 The City Drive South Orange, Irvine, CA 92868 USA; 2 University of California, San Diego Moores Cancer Center, 3855 Health Sciences Drive, La Jolla, CA 92029-S0987 USA

**Keywords:** ARID1A, EZH2, Targeted therapy, Molecular carcinogenesis, Synthetic lethality

## Abstract

Clear cell carcinoma and endometrioid adenocarcinoma are histologic subtypes of ovarian and uterine cancer that demonstrate unique clinical behavior but share common underlying genomic aberrations and oncogenic pathways. *ARID1A* mutations are more frequently identified in these tumors, in comparison to other gynecologic histologies, and loss of *ARID1A* tumor suppressor function is thought to be an essential component of carcinogenic transformation. Several therapeutic targets in *ARID1A* mutated cancers are in development, including EZH2 inhibitors. EZH2 facilitates epigenetic methylation to modulate gene expression, and both uterine and ovarian cancers show evidence of EZH2 over expression. EZH2 inhibition in *ARID1A* mutated tumors acts in a synthetically lethal manner to suppress cell growth and promote apoptosis, revealing a unique new therapeutic opportunity. Several phase 1 and 2 clinical trials of EZH2 inhibitors are ongoing currently and there is considerable promise in translational trials for utilization of this new targeted therapy, both to capitalize on *ARID1A* loss of function and to increase sensitivity to platinum-based adjuvant chemotherapies. This review will synthesize the molecular carcinogenesis of these malignancies and their unique clinical behavior, as a foundation for an emerging frontier of targeted therapeutics – the synergistic inhibition of EZH2 in *ARID1A* mutated cancers.

## Introduction

Ovarian and uterine cancer represent gynecologic malignancies with significant morbidity and mortality in the advanced and recurrent settings. Clear cell carcinoma (CCC) and endometrioid adenocarcinoma are histologic subtypes of both ovarian and uterine cancer that demonstrate unique clinical behavior, although emerging literature suggests that they may share underlying genomic aberrations and oncogenic pathways. This review explores the evolving paradigm of targeted therapeutics, as the understanding of the pathogenesis of these cancers becomes paramount to exploring new therapeutic frontiers and achieving improved oncologic outcomes.

### Low grade endometrioid and clear cell uterine carcinoma

Endometrial cancer is the most common gynecologic malignancy, with 61,380 estimated new cases and 10,920 estimated deaths in 2017 [1]. Approximately 75% of women are diagnosed with stage I disease, confined to the uterus, and have excellent 5-year survival [2]. While the majority of patients are diagnosed with well differentiated, International Federation of Gynecology and Obstetrics (FIGO) grade 1 and 2 endometrioid adenocarcinoma, a subset of patients have estrogen-independent histologies, including grade 3 endometrioid adenocarcinoma, serous carcinoma, clear cell carcinoma, carcinosarcoma, uterine sarcomas and undifferentiated cell types.

Clear cell endometrial carcinoma is rare, accounting for 1–6% of all endometrial cancers. Endometrial CCC is more likely to present with extra-uterine spread compared to low grade endometrioid histologies and is an independent predictor of poor prognosis. The proportion of patients with FIGO stage III-IV disease at the time of diagnosis is 36% with a 5-year disease specific survival of 68% [2]. Given its rarity, optimal management strategies are not well defined and treatment is extrapolated from large studies primarily comprised of the more common endometrioid histology. Management of endometrial CCC includes comprehensive surgical staging and typically adjuvant platinum-based chemotherapy and/or radiotherapy.

### Endometrioid and clear cell ovarian carcinoma

Ovarian cancer, while less common than uterine cancer, is generally diagnosed at a more advanced stage, resulting in compromised long term outcomes, with an estimated 5-year survival of 46%. In 2017 there were an estimated 22, 440 new cases and 14, 080 deaths [1]. Ovarian cancers are broadly categorized by origin, into epithelial and non-epithelial neoplasms. The most common epithelial histology, high grade serous carcinoma, accounts for nearly 70% of ovarian cancers, and is disproportionately represented in clinical trials exploring novel therapeutic paradigms. In addition to high grade serous histology, epithelial ovarian cancers include endometrioid, clear cell, mucinous, carcinosarcoma, mixed epithelial and undifferentiated.

Ovarian endometrioid carcinoma accounts for approximately 10% of epithelial ovarian cancers. Clinically, these often present with disease confined to the pelvis, may be bilateral in up to 28% of cases, and are generally low grade and early stage, portending a more favorable prognosis when compared to high grade serous carcinoma [3]. Approximately 20% of patients with ovarian endometrioid carcinomas will also have simultaneous endometrial adenocarcinomas, and both histologies are thought to share molecular aberrations [4, 5]. Furthermore, endometriosis is highly prevalent in patients with endometrioid ovarian cancer, reported in up to 35.9% of cases [6]. Management paradigms are analogous to the more common high grade serous histology, and include surgical cytoreduction followed by adjuvant platinum based combination chemotherapy [7].

Ovarian CCC constitutes approximately 5–10% of all ovarian malignancies. Although commonly identified at an early stage, with ovarian confined disease, patients may be diagnosed with advanced stage disease, or suffer recurrence, both of which are difficult to treat given the relative resistance of this histology to standard chemotherapy [8]. When compared to high grade serous carcinoma, stage-for-stage survival is lower in patients with ovarian CCC [8]. As discussed above, treatment is dependent on surgical cytoreduction followed by combination platinum-based cytotoxic chemotherapy for patients with stage 1C or greater disease.

Given the rarity of these histologies as well as their unique characteristics, additional therapeutic strategies are urgently needed to improve survival in patients suffering from advanced stage or recurrent endometrioid and clear cell ovarian cancer.

## Review

### What is *ARID1A*?

Gene expression relies on the complex interplay of transcription factors, cofactors and chromatin regulators. The role of transcription dysregulation as a mechanism for cancer is well established. With the emergence of genome-wide analyses, the significant role of chromatin remodeling complexes on gene expression levels through modified transcription, replication, repair, recombination and methylation of DNA has been identified [9]. One such complex is the Switch/Sucrose Non-Fermentable (SWI/SNF) complex which is involved in activation and inhibition of transcription. The SWI/SNF complex plays a unique role in carcinogenesis and may be mutated in over 20% of human cancers [10]. *ARID1A*, an acronym for the gene AT-rich interacting domain-containing protein 1A, encodes a component of the SWI/SNF complex and has high mutation rates across multiple malignancies [11]. Mutations are typically frameshift or nonsense mutations, often occurring at either the nuclear-export signal sequence or at the *ARID1A* interaction site with the SWI/SNF complex resulting in protein complex instability [12]. This complex modifies expression of multiple genes, including p53, through direct interaction, SMAD3, CDKN1A (p21), MLH1 and PIK3IP1 through transcription regulation of downstream effectors, and transformation of cells through the PI3K/AKT pathway [13].

### *ARID1A* as a tumor suppressor gene

*ARID1A* mutations typically results in loss of protein function with implications for cell proliferation, differentiation and apoptosis – essential roles of a tumor suppressor gene. Initial efforts into understanding its role in tumorigenesis began approximately a decade ago. The knockout of *ARID1A* in embryonic stem cells resulted in loss of self-renewal properties and substantially modified cellular differentiation [14]. In *ARID1A* knockout leukemia cell populations, Fas-mediated cell death is inhibited, supporting altered apoptosis [11, 15]. In 2011, Guan et al. restored wild-type *ARID1A* expression in ovarian cancer cells harboring deleterious mutations and noted reactivation of protein function. Additional work by Guan et al. in 2014 utilizing xenograft models confirmed that silencing *ARID1A* expression in nontransformed cells promoted cellular proliferation [13, 16]. These cumulative efforts were essential in establishing the role of *ARID1A* as a tumor suppressor gene.

### *ARID1A* in ovarian cancers

As the molecular characterization of solid malignancies expanded, it became evident that *ARID1A* mutations were most pronounced in gynecologic cancers. Within ovarian cancer cohorts, mutation rates of 46–57% were identified in clear cell adenocarcinoma and 30% in endometrioid adenocarcinoma [17, 18]. This is contrasted with the absence of identifiable *ARID1A* mutations in high-grade serous carcinomas.

Endometrioid and clear cell ovarian carcinomas are uniquely associated with endometriosis, and have been recently referred to as endometriosis-associated ovarian cancer (EAOC). Between 14 and 42% of endometrioid and 20–36% of clear cell carcinomas are associated with endometriotic lesions or patient reported symptoms of endometriosis [6, 19]. Common genetic alterations, particularly in *ARID1A*, have been recognized within atypical endometriosis and adenocarcinoma, suggesting a significant carcinogenic role [20, 21]. Loss of *ARID1A* is more frequent in endometriosis associated neoplasms, with loss of *ARID1A* immunohistochemical expression in 61% of endometriosis associated clear cell carcinomas [22]. While it is unclear if *ARID1A* mutations alone are sufficient to induce cancer progression, concurrent mutations in alternate pathways, including the PI3K/AKT pathway are frequent and appear to occur simultaneously to facilitate tumorigenesis [22, 23].

### *ARID1A* in endometrial cancers

*ARID1A* mutations are also found frequently among women with endometrial cancers, with mutation frequency of 40% in uterine endometrioid adenocarcinoma [24]. Low grade endometrioid carcinoma is the most predominant histology, with 29% of grade 1 and 2 tumors showing loss of expression, in contrast to 39% with grade 3 tumors, 26% of uterine clear cell carcinomas, and 18% of uterine serous carcinomas [25]. The Cancer Genome Atlas further confirmed that *ARID1A* is mutated in a high proportion of type I uterine cancers, particularly in the POLE hypermutated, microsatellite unstable (MSI) hypermutated, and copy-number low subgroups. These often occurred concurrently with inactivating PTEN mutations [26].

### *ARID1A* as a biomarker

**Fig. 1:**
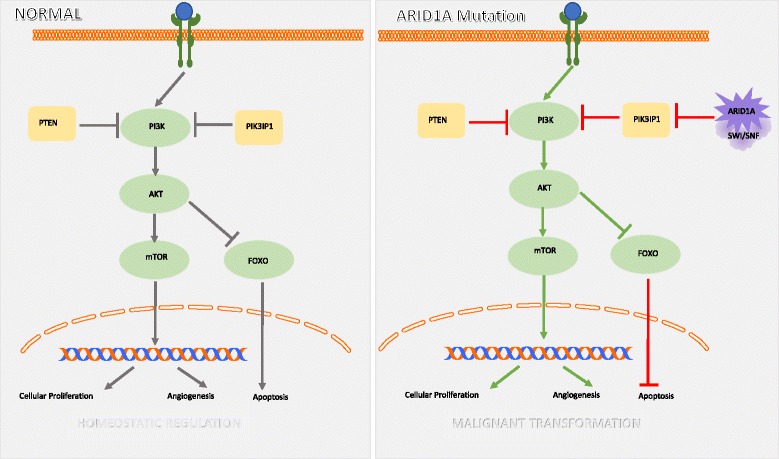
Normal and *ARID1A* cellular pathways. *ARID1A* mutation disrupts the homeostatic mechanisms of the PI3K/AKT/mTOR pathway, resulting in cellular proliferation, angiogenesis and inhibited apoptosis (Fig. 1)

Given the heterogeneity of gynecologic cancers, a consistent relationship between the presence of an *ARID1A* mutation and prognosis has been elusive. In a 2015 meta-analysis of 5651 patients with a variety of tumor types, *ARID1A* was evaluated via genetic analysis and immunohistochemistry with findings that *ARID1A* deficient tumors had significantly increased cancer-specific mortality (HR = 2.55) and cancer recurrence (HR = 1.93) when compared to a matched *ARID1A* positive population [27]. Conversely, in patient cohorts with high grade endometrioid and clear cell endometrial cancer, *ARID1A* mutations were not associated with clinical stage, depth of myometrial invasion, lymph node metastasis, or overall survival [28–30]. In a separate study, Yokoyama et al. found that *ARID1A* expression levels using immunohistochemistry correlated with prognosis and chemoresistance in stage III and IV epithelial ovarian cancers. Women with low *ARID1A* expression more frequently had incomplete response to chemotherapy (*p* = 0.026) and were more likely to experience relapse after achieving a complete response (*p* = 0.07). *ARID1A*-negative tumors had significantly worse progression free survival than *ARID1A*-positive tumors [31]. Additionally, Itamochi et al. confirmed an association between *ARID1A* immunohistochemical expression and FIGO stage as well as prognosis, with stage I and II patients having 91% 5-year survival with normal *ARID1A* expression and 74% 5-year survival with negative *ARID1A* expression [32] (Fig. 1).

### Potential molecular therapeutic targets in *ARID1A* mutated cancers

**Fig. 2:**
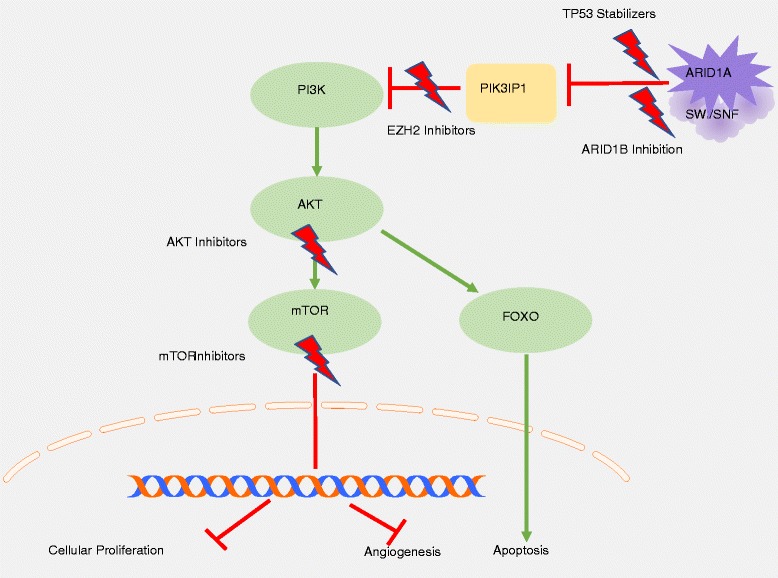
Therapeutic targets in *ARID1A* mutated cancers. Novel targeting of several key regulators in the PI3K/AKT/mTOR pathway and SWI/SNF complex modulate downstream effectors to inhibit cellular proliferation and promote apoptosis (Fig. 2)

Loss of *ARID1A* function facilitates tumorigenesis through its chromatin-mediated dysregulation of gene expression and loss of tumor suppressor gene function. As *ARID1A* plays an integral role in cell-cycle control and DNA damage repair pathways, the loss of *ARID1A* function with concurrent dysfunction in p53, p21, MLH1, or the PI3K/AKT/mTOR pathway allow malignant progression, and have revealed several potential therapeutic targets in *ARID1A* mutated cancers. These are detailed in Table 1 and Fig. 2 and several are being actively investigated for the treatment of epithelial ovarian cancer.

**Table 1:** Therapeutic targets being explored in *ARID1A* mutated cancers

EZH-2	Epigenetic synthetic lethality, promotion of apoptosis	GSK126	[33, 34]
	PIK3IP1 mediated inhibition of PI3K/AKT pathway		
mTOR	Inhibition of downstream regulator of PI3K/AKT pathway	Temsirolimus	[35, 36]
		Ridaforolimuus	
		Everolimus	
		AP23573	
TP53	Stabilization of wild-type p53 to overcome ARID1A loss, resume tumor suppressor function	Nutlin 3	[13, 37]
PI3K/AKT	Inhibit upregulated AKT phosphorylation caused by concurrent mutations	Sorafenib	[38, 39]
		Copanlisib	[36]
		BKM120	
		XL147	
BRCA	Enhance DNA-damaging effects of platinum chemotherapies EZH2 modulated function of BRCA1		[40, 41]
ARID1B	Inhibition of residual AWI/SNF complex to suppress cell growth		[42, 43]
Anti-IL6	Inhibit inflammatory microenvironment and escape from anti-tumor immune response		[44]

### The role of EZH2 in cancer

Functional studies of ovarian cancer cell lines reveal numerous gene targets of the SWI/SNF complex which may be impacted by *ARID1A* mutation, including cyclins, c-myc, and the Polycomb complexes [33, 34]. The enhancer of zeste homolog 2 (EZH2) protein is the enzymatically active core of the polycomb repressive complex 2/3 (PRC2), responsible for trimethylation of lysine 27 of histone H3 (H3K27) and can induce gene suppression through promoter binding [35, 36]. EZH2 plays a role in cancer progression through several mechanisms, including gain-of-function and loss-of-function mutations, overexpression of EZH2, mutations in the H3K27 demethylase gene, and through antagonistic mutations in the SWI/SNF chromatin remodeling complex. Within the gynecologic cancer arena, the predominant mechanisms are EZH2 overexpression and SWI/SNF antagonism of the Polycomb complex. EZH2 overexpression manifests through histone hypermethylation, resulting in tumor proliferation, cell cycle dysregulation, metastatic spread, and angiogenesis [37–39].

### EZH2 in ovarian cancers

EZH2 overexpression is found in 50–85% of ovarian carcinomas, with high expression correlating with high grade, more advanced stage disease and poor survival [40, 41]. EZH2 plays an integral role in cellular proliferation, apoptosis and invasion in human epithelial ovarian cancer cell lines ([39, 42]. EZH2 is often overexpressed in ovarian clear cell carcinomas [42]. Additionally, these tumors are characterized by genomic instability and thus, epigenetic modification of gene expression may play a critical role in tumorigenesis.

### EZH2 in uterine cancers

EZH2 targets genes which may exhibit modified function in endometrial cancer including: p16, E-cadherin, SFRP1, DKK3, and β-catenin [43–45]. EZH2 overexpression was identified in complex hyperplasia, atypical hyperplasia, and endometrial carcinoma but not in simple hyperplasia or normal endometrial tissue [45]. Overexpression has been associated with high proliferation rates and aggressive tumor subgroups of endometrial cancers ([43, 44]). EZH2 expression correlated with high tumor grade, deep myometrial invasion, lymphovascular space invasion and enhanced cellular proliferation, as well as decreased overall survival, suggesting a role as both a prognostic and therapeutic marker in endometrial cancer [44–46].

### Synthetic lethality

Given the reversible epigenetic modifications which drive tumorigenesis, EZH2 methyltransferase activity is an ideal target for cancer therapeutics. Several new selective small molecules targeting EZH2 have been developed, including GSK126 [47], EPZ005687 [48] and EI1 [49], all of which inhibit EZH2 without otherwise effecting the PCR2 complex.

**Fig. 3:**
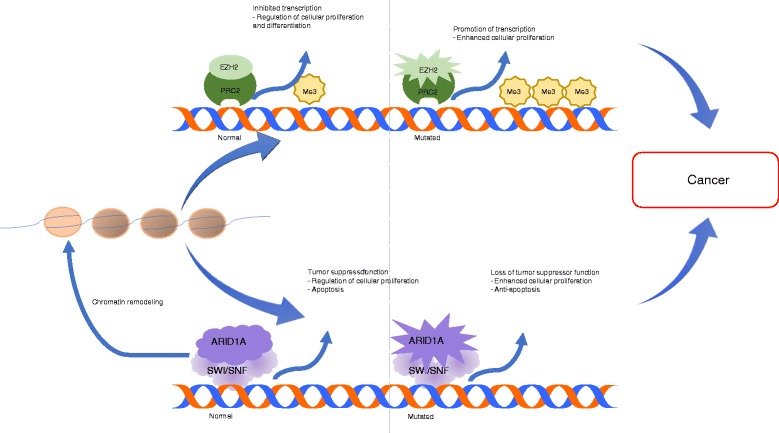
Mechanisms of both normal and mutated *ARID1A* and EZH2. Homeostasis requires balanced *ARID1A* and EZH2 function, while malignant transformation arises with dysregulation of either process (Fig. 3)

**Fig. 4:**
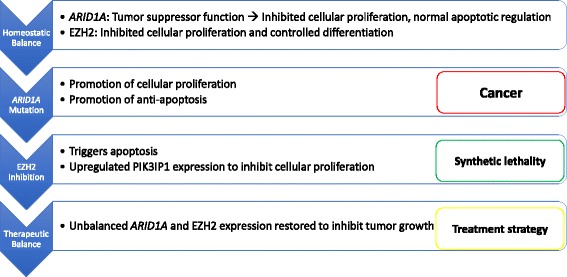
Mechanisms of EZH2 inhibitor synthetic lethality in *ARID1A* mutated tumors. Balanced action of EZH2 and *ARID1A* are necessary for normal cellular function

Homeostasis requires balanced action of *ARID1A* and EZH2 through chromatin-mediated gene expression. Loss of *ARID1A* expression results in imbalanced EZH2 activity, and is hypothesized to drive tumorigenesis (Figs. 3 and 4). Mechanistically, both *ARID1A* and EZH2 target PI3K-interacting protein 1 gene (PIK3IP1), with resultant silencing causing cell proliferation and promotion of anti-apoptotic effects through the PI3K-AKT pathway. In a pivotal study by Bitler et al., it was demonstrated that targeted EZH2 inhibition triggers apoptosis in *ARID1A* mutated cells and upregulates PIK3IP1 expression, thereby suppressing cell growth [50]. This cooperation of *ARID1A* mutation and EZH2 targeted inhibition represents a synthetically lethal interaction. This is particularly exciting in that drug-gene synthetic lethality often allows utilization of low concentrations of drugs, minimal toxicity, and limited treatment resistance (Table 2).

**Table 2:** Ongoing EZH2 targeted clinical trials

Targeted Agent	Trade Name	Route	Trial	Tumor	Status/Adverse Events (Grade 3–4)	References
EPZ-6438	Tazemetostat	PO	Phase 1	NHL	Thrombocytopenia Neutropenia Hypertension Anorexia Transaminitis	[58]
		PO	Phase 1/2	B-cell lymphoma or advanced solid tumor	Recruiting actively	(Clinicaltrials.Gov. (2.13.2017)), (Epizyme pharmaceuticals website. (2.13.2017))
					NCT01897571	
					Nausea	
					Asthenia	
					Thrombocytopenia	
					Neutropenia	
					Fatigue	
		PO	Phase 1	Pediatric INI1 negative tumors or synovial sarcoma	Recruiting actively	(Clinicaltrials.Gov. (2.13.2017)), (Epizyme pharmaceuticals website. (2.13.2017))
					NCT02601937	
		PO	Phase 2	Adult INI1 negative tumors or synovial sarcoma	Recruiting actively	(Clinicaltrials.Gov. (2.13.2017)), (Epizyme pharmaceuticals website. (2.13.2017))
					NCT02601950	
		PO	Phase 2	Malignant mesothelioma	Recruiting actively	(Clinicaltrials.Gov. (2.13.2017)), (Epizyme pharmaceuticals website. (2.13.2017))
					NCT02860286	
GSK 2,816,126	GSK126	IV	Phase 1/2	Diffuse large B-cell lymphoma	Fatigue	[59]
					Nausea	
					Vomiting	
					Anemia	
EPZ-5676	Pinometostat	IV	Phase 1	Leukemia	Hypophosphatemia	[60]
					Neutropenia	
					Reduced ejection fraction	
					Transaminitis	
CPI-1205		IV	Phase 1	B cell lymphoma	Recruiting actively	(Clinicaltrials.Gov. (2.13.2017)),
					NCT02395601	

### EZH2 targeted therapeutics

GSK126 was shown to be well tolerated in mouse xenograft models of ovarian CCC [47]. Of numerous available targeted small molecules, GSK126 exhibited the highest sensitivity against *ARID1A* deficient ovarian CCC cells [50]. Bitler et al. showed that GSK126 significantly decreased tumor burden and reduced the number of metastatic peritoneal tumor implants in a mouse clear cell ovarian xenograft model when compared to controls. On a mechanistic level, immunohistochemical analysis showed decreased H3K27Me3 levels, increased PIK3IP1 activity, as well as cleaved caspase 3 in the GSK126 treated mice. This further confirmed the PIK3PI1 shared gene target, allowing synthetic lethality in *ARID1A*-deficient tumors via EZH2 inhibition [50].

### Resensitization to platinum based chemotherapies

In advanced clear cell ovarian carcinoma, approximately half of patients progress while on platinum-based chemotherapy, in comparison to 29% of those with high grade serous histology. Additionally, patients with advanced stage clear cell carcinoma have compromised survival outcomes, with a median overall survival overall of approximately 12 months, significantly less than those with high grade serous histology [51]. This compromised outcome in patients with clear cell carcinoma is thought to result from relative platinum resistance, and thus mechanisms to increase platinum sensitivity may significantly impact morbidity and mortality in this subset of patients. EZH2 downregulation in ovarian cancer has been shown to sensitize tumor cells to cisplatin and EZH2 overexpression is associated with resistance to cisplatin through H3K27 tri-methylation of drug-resistance genes [52, 53].

Translational research suggests that combination therapies utilizing platinum-based cytotoxic chemotherapy and EZH2 inhibitors, may be particularly potent and synergistic in ARID1A mutated tumors [54]. Within non-Hodgkin’s lymphoma tumors with EZH2 mutations, a combination of EPZ-6438 and traditional targeted chemotherapy prevented tumor growth [55]. Prostate cancer cell lines had increased tumor death when treated with combined etoposide and GSK126, again suggesting synergistic therapeutic effect [56]. Interestingly, EZH2 inhibition had varying effects in a preclinical study of non-small cell lung cancers, with increased sensitization to topoisomerase II inhibitors in the tumor subset demonstrating BRG1/SMARCA4 loss-of-function mutations or EGFR gain-of-function mutations [57]. These findings in non-gynecologic tumors provide a foundation for exploring the cooperative effects of chemotherapy and EZH2 inhibition within ovarian and uterine malignancies.

### Future directions

The potential therapeutic role of EZH2 inhibition in *ARID1A* mutated gynecologic cancers may represent a novel and exciting treatment paradigm in a subset of patients with limited treatment options. Capitalizing on the concept of synthetic lethality induced in this population using EZH2 inhibitors, as well as the potential synergistic effects with platinum-based chemotherapies, may help translate into improved oncologic outcomes.

Given the frequency of *ARID1A* mutations in patients with clear cell and endometrioid ovarian and uterine cancer, and the prognostic implications of loss of *ARID1A* expression, it is natural to explore the impact of EZH2 inhibition in these patient subsets. Given the above, clinical trialists are currently designing a basket trial concept of the EZH2 inhibitor, Tazemetostat, in patients with recurrent, measurable, clear cell and endometrioid ovarian cancer, as well as recurrent endometrioid endometrial cancer. In this study, subjects with recurrent, measurable disease, will be enrolled and treated with single agent Tazemetostat at the recommended phase 2 dose of 800 mg orally twice daily until disease progression or unacceptable toxicity. *ARID1A* mutation status will be an integrated biomarker, and will be assessed via BAF250a IHC, as well as next generation sequencing, allowing investigators to determine the correlation between *ARID1A* mutation status and IHC expression. Furthermore, sequencing studies will facilitate assessment of the mutation status of all other SWI/SNF members.

Ultimately, if single agent regimens using EZH2 inhibitors are shown to be effective in this subset of patients, then novel combinatorial approaches utilizing platinum-based chemotherapy, TP53 stabilizers, PI3K/AKT inhibitors, or mTOR inhibitors may be warranted.

## Conclusions

In an effort to expand the therapeutic portfolio for patients with advanced stage and recurrent ovarian and endometrial cancer, molecular characterization of theses lesions has emerged as a clinical priority. Using both institutional genomic data, and data from the NCI sponsored cancer genome atlas, *ARID1A* mutations were frequently identified as mutated tumor suppressor genes, in clear cell and endometrioid ovarian cancer as well as low grade endometrioid endometrial cancer. Ultimately, clinicians will look to capitalize on this molecular aberration via novel targeted therapies, such as tazemetostat, as single agents or in combination regimens. As our understanding of the molecular mechanisms underlying malignant transformation evolves, the discovery of novel targeted therapies that lead to meaningful survival gains may become a reality.

## Abbreviations

ARID1A: AT-rich interacting domain-containing protein 1A
CCC: Clear cell carcinoma
EZH2: Enhancer of zeste homolog 2
FIGO: International Federation of Gynecology and Obstetrics
PRC2: Polycomb repressive complex 2/3
SWI/SNF: Switch/Sucrose Non-Fermentable
